# Silibinin induces mitochondrial NOX4-mediated endoplasmic reticulum stress response and its subsequent apoptosis

**DOI:** 10.1186/s12885-016-2516-6

**Published:** 2016-07-12

**Authors:** Sang-Hun Kim, Kwang-Youn Kim, Sun-Nyoung Yu, Young-Kyo Seo, Sung-Sik Chun, Hak-Sun Yu, Soon-Cheol Ahn

**Affiliations:** Department of Microbiology & Immunology, Pusan National University School of Medicine, Yangsan, 626-870 Republic of Korea; School of Life Sciences, Ulsan National Institute of Science and Technology, Ulsan, 689-798 Republic of Korea; Immunoregulatory Therapeutics Group in Brain Busan 21 Project, Pusan National University, Yangsan, 626-870 Republic of Korea; Department of Food Science, International University of Korea, Jinju, 660-759 Republic of Korea; Department of Parasitology, Pusan National University School of Medicine, Yangsan, 626-870 Republic of Korea

**Keywords:** Silibinin, Apoptosis, Reactive oxygen species, NOX, Ca^2+^, Endoplasmic reticulum stress

## Abstract

**Background:**

Silibinin, a biologically active compound of milk thistle, has chemopreventive effects on cancer cell lines. Recently it was reported that silibinin inhibited tumor growth through activation of the apoptotic signaling pathway. Although various evidences showed multiple signaling pathways of silibinin in apoptosis, there were no reports to address the clear mechanism of ROS-mediated pathway in prostate cancer PC-3 cells. Several studies suggested that reactive oxygen species (ROS) play an important role in various signaling cascades, but the primary source of ROS was currently unclear.

**Methods:**

The effect of silibinin was investigated on cell growth of prostate cell lines by MTT assay. We examined whether silibinin induced apoptosis through production of ROS using flow cytometry. Expression of apoptosis-, endoplasmic reticulum (ER)-related protein and gene were determined by western blotting and RT-PCR, respectively.

**Results:**

Results showed that silibinin triggered mitochondrial ROS production through NOX4 expression and finally led to induce apoptosis. In addition, mitochondrial ROS caused ER stress through disruption of Ca^2+^ homeostasis. Co-treatment of ROS inhibitor reduced the silibinin-induced apoptosis through the inhibition of NOX4 expression, resulting in reduction of both Ca^2+^ level and ER stress response.

**Conclusions:**

Taken together, silibinin induced mitochondrial ROS-dependent apoptosis through NOX4, which is associated with disruption of Ca^2+^ homeostasis and ER stress response. Therefore, the regulation of NOX4, mitochondrial ROS producer, could be a potential target for the treatment of prostate cancer.

**Electronic supplementary material:**

The online version of this article (doi:10.1186/s12885-016-2516-6) contains supplementary material, which is available to authorized users.

## Background

Reactive oxygen species (ROS) can act as secondary messengers in cancer cell signaling. It plays an important role in various cellular response, including cell growth, differentiation, survival, death, inflammation and immune response [1, 2]. The sources of ROS are various organelles and enzymes system including mitochondria, endoplasmic reticulum, peroxisomes and NADPH oxidase (NOX) [3]. ROS are generated in forms of superoxide anion (O_2_–•), hydroxyl radical (OH•) and hydrogen peroxide (H_2_O_2_) in living organisms [4]. Most of O_2_–• are produced by NOXs, xanthine oxidase and the mitochondrial electron-transport chain [5]. Extracellular stresses including toxin, growth factors, ROS, ultraviolet radiation, viral infection and anti-cancer agents are known to trigger apoptosis in many studies [6–9]. In addition, apoptosis occurs from the changes of mitochondrial membrane potential (MMP, *ΔΨ*_*m*_) and release of pro-apoptotic factor such as cytochrome c [10]. ROS are critically important signals to activate endoplasmic reticulum (ER) stress as well as apoptosis by a variety of stimulating conditions [11].

Recently, several studies have reported that the apoptosis is related with ER stress responses in cancer cell lines [11, 12]. ER stress is involved in the initiation of apoptosis by at least two different mechanisms, namely the unfolded protein response (UPR) and Ca^2+^ signaling [13, 14]. The UPR-mediated signals consist of a complex interaction between three signaling, inositol requiring enzyme 1 (IRE1)-X box-bonding protein 1 (XBP1) signaling, pancreatic ER kinase (PKR)-like ER kinase (PERK)-eukaryotic initiation factor 2α (eIF2α) signaling and activating transcription factor 6 (ATF6) signaling [15]. The activation of ER membrane-resident caspase-4/12 (human/mice) and induction of CHOP stimulate ER stress-mediated apoptosis [16, 17]. Subsequently, the typical signaling of apoptosis mainly lead to the activation of intracellular caspases, major activator of the mitochondrial-dependent pathway [18]. ER is the main intracellular storage compartment for Ca^2+^, which is an important secondary messenger required for cellular functions. Changes of cellular Ca^2+^ homeostasis including cytosolic Ca^2+^ overload, ER Ca^2+^ depletion and mitochondrial Ca^2+^ increase induce apoptosis [19, 20]. The ER-mitochondria interaction supports communication between the two organelles, including the exchange of Ca^2+^, which controls ER chaperone protein, mitochondrial ATP production and apoptosis [21].

Silibinin, a major biologically active constituent of silymarin from milk thistle extract, has been used clinically for its hepatoprotective action for more than three decades in Europe and recently in Asia and the United States [22]. It has been reported that it induces apoptosis by regulating the cell cycle arrest in human bladder carcinoma cells and human colon carcinoma HT-29 cell [23, 24]. Other reports in murine orthotopic hepatocarcinoma model have indicated that it inhibits tumor growth through the activation of TRAIL/death receptor/apoptotic signaling pathway, both in vitro and in vivo [25]. In addition, it has been shown anti-tumor effect in which induces apoptosis through a p53-dependent pathway involving Bcl-2/Bax, cytochrome c release and caspase activation [26]. So far, it has not been clarified that production of ROS or Ca^2+^ signaling-mediated ER stress play a critical role in the silibinin-induced apoptosis in human prostate cancer PC-3 cells. Therefore, we investigated whether production of ROS by silibinin causes ER stress through changes of Ca^2+^ concentration and whether these intracellular signaling pathways lead to cellular apoptosis.

## Methods

### Reagents and antibodies

3-(4,5-Dimethyl-thiazol-2-yl)-2,5-diphenyltertrazolium bromide (MTT), propidium iodide (PI), 6-diamidino-2-phenylindole dihydrochloride (DAPI), 2′,7′-dichlorfluorescein-diacetate (DCFH-DA) and 4-(6-Acetoxymethoxy-2,7-dichloro-3-oxo-9-xanthenyl)-4′-methyl-2,2′(ethylenedioxy)dianiline-N,N,N′,N′-tetraacetic acid tetrakis (acetoxymethyl) ester (Fluo-3/AM) were purchased from Sigma Chemical Co. (St. Louis, MO, USA). 1,2-bis-(o-Aminophenoxy) ethane-tetraacetic acid tetra-(acetoxymethyl) ester (BAPTA/AM), Diphenyleneiodonium (DPI), Z-DEVD-FMK and Z-YVAD-FMK were purchased from Calbiochem (Merck, Darmstadt, Germany) and R&D Systems (Minneapolis, MN, USA), respectively. FITC Annexin-V apoptosis Detection kit and Caspase-3 Colorimetric Assay Kit were purchased from BD Bioscience (San Jose, CA, USA) and Assay Design Inc. (Ann Arbor, Michigan, USA), respectively. RiboEx_column™ kit was purchased from GeneAll Biotechnology Co. (Seoul, Korea). TOPscript™ RT DryMIX kit (Enzynomics, Deajeon, Korea) and EmeraldAmp PCR masterMIX (TAKARA, Otsu, Japan) were obtained. The ECL Western Kit was purchased from iNtRON Biotechnology (Seongnam, Korea). Antibodies for NOX4, Caspase-3 and β-Actin were purchased from Santa Cruz Biotechnology (Dallas, TX, USA). Antibodies for poly (ADP-ribose) polymerase-1 (PARP-1), Bip, IRE1α, and CHOP were purchased from Cell Signaling (Beverly, MA, USA). MitoSOX was purchased from Invitrogen (Grand Island, NY, USA).

### Cell lines and cell culture

Human prostate cell lines, PC-3, LNCaP and RWPE-1 cells were obtained from the American Type Culture Collection (ATCC, Manassas, VA, USA). Prostate cancer PC-3 and LNCaP cells were cultured in Dulbecco’s modified Eagle’s minimal medium (DMEM, WelGENE Inc., Korea) and Roswell Park Memorial Institute (RPMI) 1640 (WelGENE Inc., Korea) supplemented with 10 % FBS and 1 % penicillin-streptomycin solution with 5 % CO_2_ at 37 °C, respectively. Prostate normal RWPE-1 cells were cultured in keratinocyte serum-free media (K-SFM) containing 2.5 μg of epidermal growth factor (EGF), 25 mg of bovine pituitary extract (BPE, GIBCO) and 1 % penicillin-streptomycin solution with 5 % CO_2_ at 37 °C.

### Cell viability assay

Cell viability was determined using the 3-(4,5-dimethylthiazol-2-yl)-2,5-diphenyltetrazolium bromide (MTT) assay. The prostate cancer PC-3 cells were seeded at a density of 1 × 10^4^/ml in a 48-well culture dish and treated with silibinin of various concentrations for 24 and 48 h. After incubation, these cells were treated with 0.5 mg/ml of the MTT solution for further 3 h incubation and the precipitates were dissolved in dimethyl sulfoxide to dissolve the MTT-formazan complex. Absorbance was recorded on a microplate reader (Molecular Devices, Sunnyvale, CA, USA) at a wavelength of 540 nm. The cell viability was determined the relatives as their percentage of the treated cells to one of the untreated cells by comparing their optical densities.

### Apoptosis assay

Apoptotic cells were quantified and analyzed using an annexin V-FITC detection kit and flow cytometry. Briefly, the PC-3 cells were treated with 150 μM of silibinin for 48 h. Then the cells were washed with phosphate buffered saline (PBS) and were collected. Harvested cells were mixed in 1X binding buffer and incubated with an annexin V/PI double staining solution at room temperature for 15 min. Then, the staining cells were analyzed by flow cytometry (FACS Calibur, Becton Dickinson, San Jose, CA, USA) and the CellQuest software (Becton Dickinson Co.).

### Measurement of MMP, Ca^2+^ flux and ROS concentration

MMP, Ca^2+^ and ROS levels were determined by the DiOC_6_, Fluo-3/AM and DCFH-DA, respectively. Briefly, silibinin-treated PC-3 cells in the presence or absence of each inhibitor were trypsinized, washed and incubated with each dye, a fluorescent marker, at 37 °C for 30 min. Fluorescence positive cells were measured by flow cytometry with CellQuest analysis software.

### Immunofluorescence confocal microscopy

The mitochondrial ROS production was measured by confocal microscopy after staining with MitoSOX (Invitrogen, Waltham, MA, USA). Briefly, PC-3 cells were seeded on coverglass bottom dish and treated with 150 μM of silibinin for 24 h. Then the cells were incubated with 5 μM MitoSOX and fixed with 4 % paraformaldehyde for 10 min at room temperature. After fixation, the cells were washed twice with PBS and then incubated with 1 μg/ml DAPI solution at 4 °C for 15 min. Images were acquired using a confocal microscope (Olympus, Tokyo, Japan). For NOX4 localization assay, PC-3 cells were stained with 5 μM MitoSOX and fixed. Cells were subsequently permeabilized with 0.1 % triton X-100 for 10 min and blocked with 5 % BSA, and incubated with NOX4 primary antibody. Cells were washed with PBS and incubated in FITC-conjugated secondary antibody for 1 h at room temperature and then stained with DAPI solution. Stained cells were visualized by an Olympus confocal microscope.

### RNA extraction and reverse transcription PCR

Total RNA was isolated using RiboEx_column™ kit according to the manufacturer’s instructions. Reverse transcription (RT) was carried out with 2 μg RNA and Oligo dT using TOPscript™ RT DryMIX kit, and the resulting cDNA was subjected to PCR. The sequence of the primers used in the PCR was the following: NOX1 (F: 5′ GTA CAA ATT CCA GTG TGC AGA CCA C 3′; R: 5′ CAG ACT GGA ATA TCG GTG ACA GCA 3′), NOX2 (F: 5′ GCT GTT CAA TGC TTG TGG CT 3′; R: 5′ TCT CCT CAT CAT GGT GCA CA 3′), NOX3 (F: 5′ GGA TCG GAG TCA CTC CCT TCG CTG 3′; R: 5′ ATG AAC ACC TCT GGG GTC AGC TGA 3′), NOX4 (F: 5′ CTC AGC GGA ATC AAT CAG CTG TG 3′; R: 5′ AGA GGA ACA CGA CAA TCA GCC TTA G 3′), NOX5 (F: 5′ TTA TGG GCT ACG TGG TAG TGG G 3′; R: 5′ GAA CCG TGT ACC CAG CCA AT 3′), XBP1 (F: 5′ CCT TGT AGT TGA GAA CCA GG 3′; R: 5′ GGG GCT TGG TAT ATA TGT GG 3′), Bip (F: 5′ TGC AGC AGG ACA TCA AGT 3′; R: 5′ CGC TGG TCA AAG TCT TCT CC 3′), CHOP (F 5′ GCG TCT AGA ATG GCA GCT GAG TCA TTG CC 3′; R: 5′ GCG TCT AGA TCA TGC TTG GTG GAG ATT C 3′), and GAPDH (F: 5′ CCA CCC ATG GCA AAT TCC ATG GCA 3′; R: 5′ GCG TCT AGA TCA TGC TTG GTG GAG ATT C 3′). The amplification was performed with EmeraldAmp PCR master MIX in Mycycler Thermal Cycler (Bio-Rad Laboratories Inc., Hercules, CA).

### Western blotting

Cell extracts were prepared by incubating the cells in lysis buffer [150 mM NaCl, 10 mM Tris (pH 7.4), 5 mM EDTA (pH 8.0), 1 % Triton X-100, 1 mM PMSF, 20 mg/ml aprotinin, 50 μg/ml leupetin, 1 mM benzamdine, 1 mg/ml pepstatin]. Forty micrograms of proteins determined by the BSA method were electrophoretically separated on 8–15 % sodium dodecyl sulfate-polyacrylamide gel electrophoresis (SDS-PAGE) and transferred to polyvinylidene fluoride (PVDF) membrane. The membranes were blocked with 5 % skim milk in TBS-T buffer [20 mM Tris (pH 7.4), 150 mM NaCl, 0.1 % Tween 20] at room temperature for 1 h. The membranes were incubated with primary and secondary antibodies and then washed 3 times with TBS-T buffer for 10 min. Finally, the proteins were detected with an ECL western blotting detection reagent. The densities of each band were determined with a fluorescence scanner (LAS 3000, Fuji Film, Tokyo, Japan) and analyzed with Multi Gauge V3.0 software.

### Statistical analysis

Experiments were repeated at least 3 times with consistent results. Unless otherwise stated, data are expressed as the mean ± SD. ANOVA was used to compare the experimental groups to the control values, whereas comparisons between multiple groups were performed using a Tukey’s multiple comparison test. The results were statistically significant at *P* <0.05.

## Results

### Silibinin stimulated ROS production from mitochondria

**Fig. 1:**
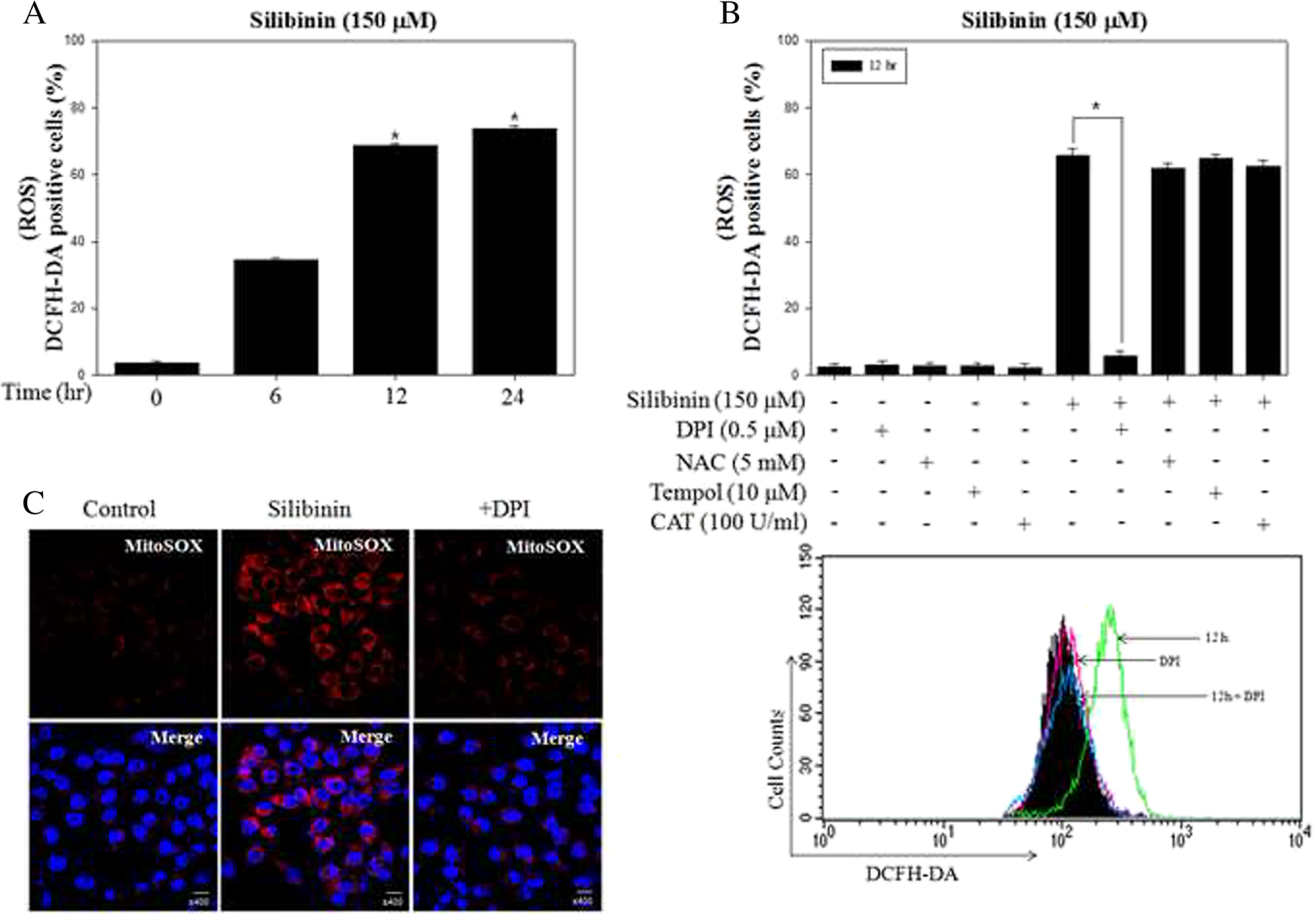
Silibinin stimulated the generation of ROS derived from mitochondria in PC-3 cells. **a** PC-3 cells were treated with 150 μM silibinin up to 24 h and ROS production was determined by the fluorescence of DCFH-DA with flow cytometry. **b** PC-3 cells were treated with 150 μM silibinin for 12 h with the presence or absence of 0.5 μM DPI, 5 mM NAC, 10 μM Tempol and 100 U/ml CAT. **c** Representative images showed mitochondrial ROS production by confocal microscopy with MitoSOX, a mitochondrial ROS dye. Data are presented as ^*^ *p* <0.001 vs. the control group

We examined the intracellular ROS involved in the initiation of apoptotic signaling, which are the byproducts of normal cellular oxidative processes [27]. As a result, intracellular ROS levels were significantly increased in a time-dependent manner in PC-3 cells treated with silibinin (Fig. 1a). Although silibinin stimulated robust ROS production up to 24 h, we confirmed viable cells by MTT assay (Additional file 1: Figure S1A). To identify the cellular source of ROS production by silibinin, PC-3 cells were treated with silibinin in the presence or absence of various ROS inhibitors (general ROS scavenger, NAC and Tempol; NOX inhibitor, DPI; H_2_O_2_ scavenger, CAT). The results showed that DPI selectively suppressed silibinin-stimulated ROS production, whereas NAC, Tempol and CAT had weak effects (Fig. 1b). These ROS were attenuated in early time stage for 3 h (Additional file 1: Figure S1B), suggesting that persistent and excessive ROS were associated with DPI-specific mechanism. DPI is known to be an inhibitor of mitochondrial ROS and NOX system. Therefore, to visualize ROS generated from mitochondria, PC-3 cells after silibinin treatment were stained with MitoSOX, a selective dye of mitochondrial-derived ROS. As a result, confocal microscopy image showed that silibinin enhanced the fluorescence intensity of MitoSOX in the mitochondrial portion and pretreatment with DPI inhibited mitochondrial ROS generation (Fig. 1c). These results suggested that silibinin stimulated ROS production from mitochondria.

### Silibinin triggered mitochondrial ROS derived from NOX4 expression

**Fig. 2:**
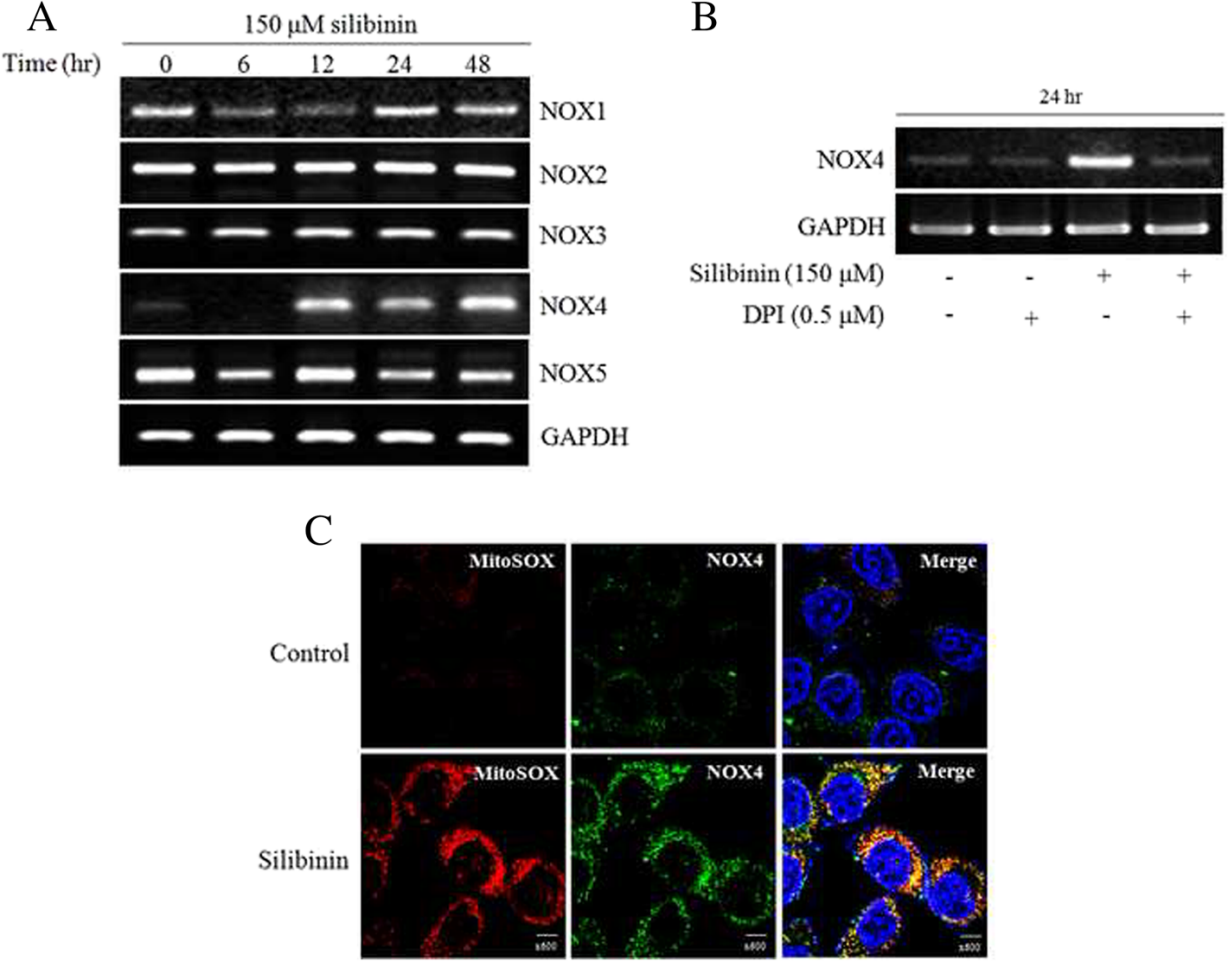
Silibinin triggered mitochondrial ROS derived from NOX4 in PC-3 cells. **a** NOX isoforms were detected by PCR using specific primers for NOX isoforms. GAPDH was used as a loading control. **b** NOX4 expression was analyzed after treatment with 150 μM silibinin for 24 h with the presence or absence of 0.5 μM DPI. **c** Representative images obtained by confocal fluorescence microscopy with MitoSOX after exposure to silibinin for 24 h. After fixation and permeabilization, it was cell stained with our Nox4 antibody and appropriate FITC-conjugated secondary antibody. Nuclei were counter-stained with DAPI

It has been suggested that NOX family members are a major source of mitochondrial ROS. A DPI has been used to inhibit ROS production mediated by NOX [28]. Thus, we confirmed the mRNA expression of various NOX isoforms after treatment of silibinin (Fig. 2a). Interestingly, among NOX isoforms, NOX4 was significantly increased by treatment of silibinin in a time-dependent manner but others expression was not changed. As expected, the increased expression of NOX4 by silibinin was suppressed by pretreatment with DPI (Fig. 2b). NOX4 is known to be localized at the mitochondrial membrane, from which stimulate ROS production [29]. Therefore, to confirm that production of mitochondrial ROS is associated with NOX4, confocal microscopy was used to visualize the intensity of fluorescence by using FITC-conjugated anti-rabbit IgG and MitoSOX. The result showed that NOX4 was colocalized with silibinin-stimulated mitochondrial ROS (Fig. 2c). Taken together, it was demonstrated that potential cellular source of mitochondrial ROS production was NOX4 system and that these ROS production may be involved in cellular apoptosis pathway.

### Silibinin induced apoptosis through mitochondrial ROS

**Fig. 3:**
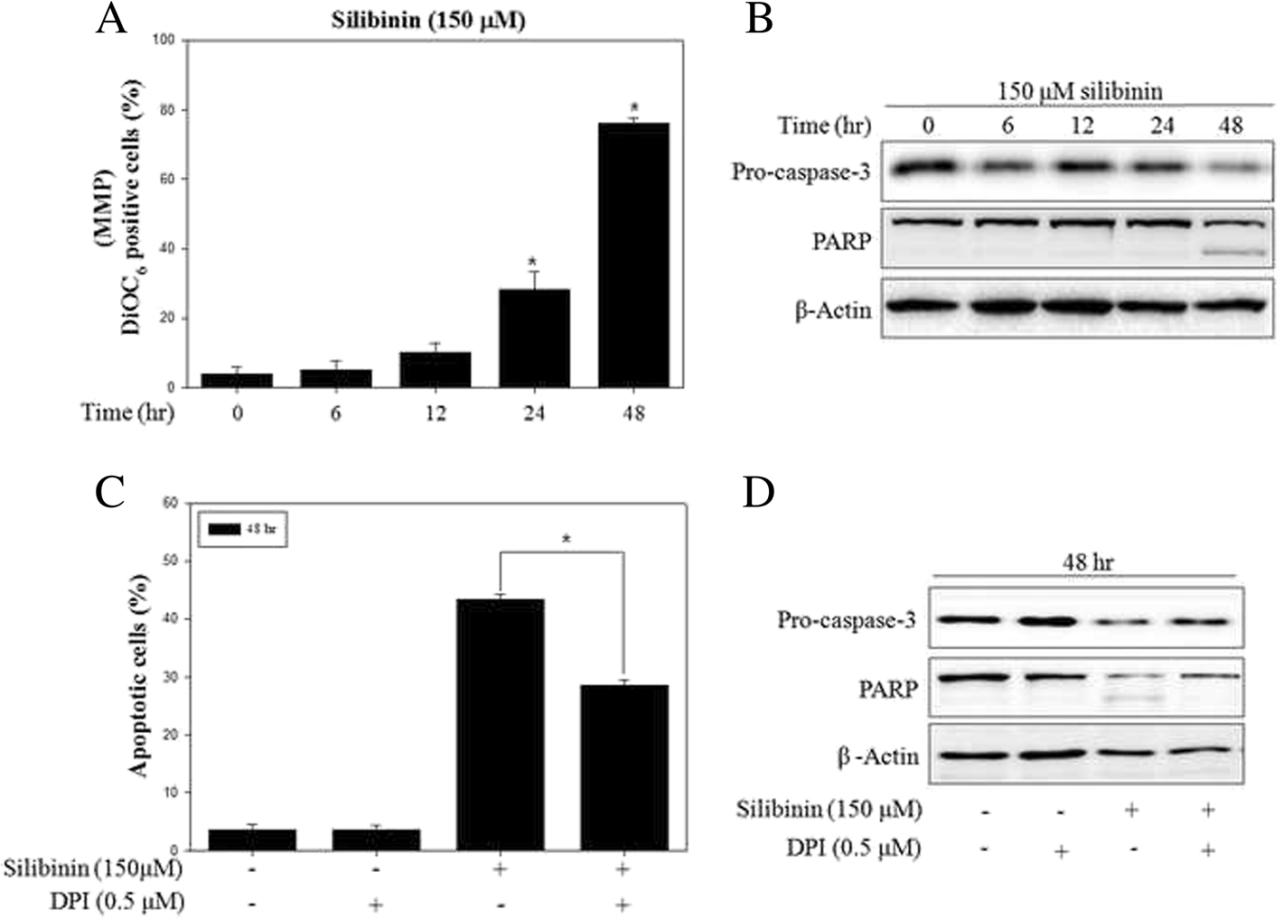
Silibinin induced apoptosis through mitochondrial ROS in PC-3 cells. **a** PC-3 cells were treated with 150 μM silibinin for indicated times. MMP was determined using fluorescence dye DiOC_6_ by flow cytometry. **b** Protein expression was analyzed by western blotting with antibodies for pro-caspase-3 and PARP. β-Actin was used as a loading control. **c** Apoptosis was analyzed after treatment with 150 μM silibinin for 48 h with the presence or absence of 0.5 μM DPI by flow cytometry. **d** Protein expression was analyzed by western blotting. β-Actin was used as a loading control. Data are presented as mean ± SD (*n* = 3 in each group). ^*^ *p* <0.001 vs. the control group

Previous studies reported that generation of intracellular ROS induce apoptosis in various cancer cells [30]. First, we confirmed evidence of apoptotic events including change of MMP and expression of apoptosis-related proteins. MMP is associated with mitochondrial function, followed by the release of cytochrome c and activation of caspase-9 and −3, which are important steps of the intracellular signaling cascade in apoptosis [31]. The effect of silibinin on MMP was analyzed using DiOC_6_, a fluorescent dye, in PC-3 cells. Changes of MMP significantly occurred after treatment with 150 μM of silibinin for up to 48 h (Fig. 3a). In addition, silibinin reduced pro-caspase-3 and increased cleavage form of PARP in a time-dependent manner (Fig. 3b), as we previously reported [32]. Next, to identify whether production of mitochondrial ROS by silibinin is associated with apoptosis, we confirmed the change of silibinin-induced apoptosis with the presence or absence of DPI. The results showed that DPI inhibited silibinin-induced apoptosis and expression of apoptosis-related proteins including caspase-3 and PARP (Fig. 3c and d). Taken together, silibinin induced apoptosis through regulation of MMP and the apoptosis is promoted from generation of mitochondrial ROS by NOX4.

### Silibinin induced ER stress through disruption of intracellular Ca^2+^ homeostasis

**Fig. 4:**
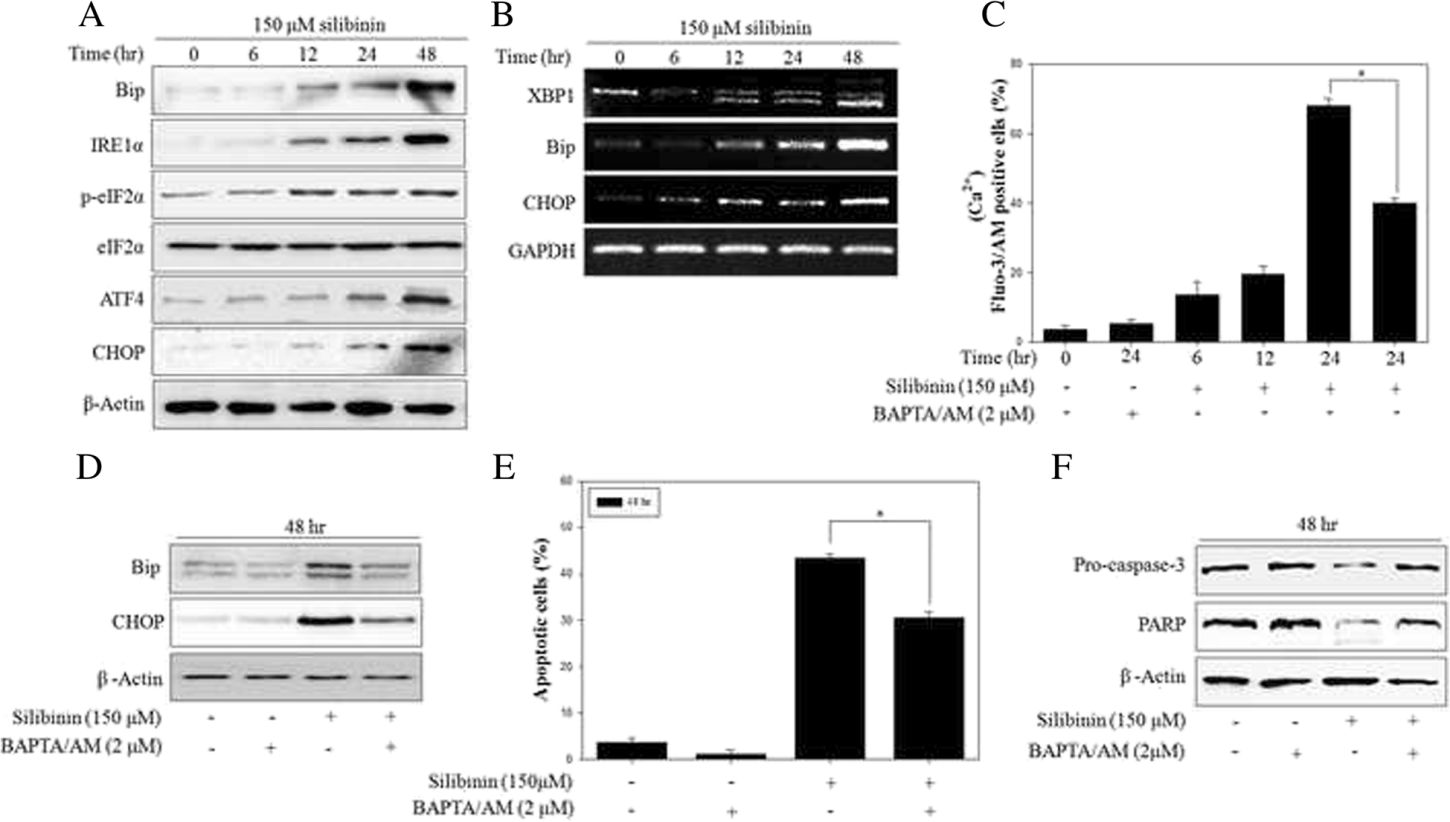
Silibinin induced ER stress response through disruption of Ca^2+^ homeostasis. **a** Expression of ER stress-related proteins was detected by western blotting with antibodies for Bip, IRE1α, p-eIF2α, eIF2α, ATF4, CHOP and β-Actin was used as a loading control. **b** ER stress-related mRNAs were detected by RT-PCR with primers for XBP1, Bip, CHOP and GAPDH was used as a loading control. **c** PC-3 cells were treated with 150 μM silibinin for 24 h with the presence or absence of 2 μM BAPTA/AM. The intracellular Ca^2+^ concentration was determined by the fluorescence of fluo-3/AM with flow cytometry. **d** Protein expression was analyzed by western blotting. β-Actin was used as a loading control. **e** Apoptosis was analyzed after treatment of 150 μM silibinin for 48 h with the presence or absence of 2 μM BAPTA/AM by flow cytometry. **f** The effects of BAPTA/AM on silibinin-induced apoptosis were measured by western blotting. β-Actin was used as a loading control. Data are presented as mean ± SD (*n* = 3 in each group). ^*^ *p* <0.001 vs. the control group

It was well established that apoptosis is regulated by ER stress response from unfolded protein accumulation [33]. To examine whether silibinin causes ER stress in PC-3 cells, we investigated Ca^2+^ homeostasis and the expression of ER stress-related proteins. The expression of Bip, IRE1α, p-eIF2α, ATF4 and CHOP, a transcription factor as pro-apoptotic factor, was time-dependently increased after silibinin treatment (Fig. 4a). The splicing form of X-box binding proteins-1 (XBP-1) mRNA level, which is a major pathway of ER stress signaling, and mRNA levels of Bip and CHOP were increased in a time-dependent manner (Fig. 4b). Because ER is main storage of the intracellular Ca^2+^, we observed whether UPR by silibinin is associated with change of Ca^2+^ flux. As a result, fluorescence intensity of Fluo-3/AM-incubated cells was continuously increased during the 24 h of silibinin treatment, indicating that silibinin elevated the level of intracellular Ca^2+^ in a time-dependent manner. These occurrences were significantly suppressed by pretreatment with BAPTA/AM, an intracellular Ca^2+^ chelator (Fig. 4c). In addition, to confirm the role of Ca^2+^ signaling in ER stress response, cells were pretreated with Ca^2+^ chelator BAPTA/AM. As a result, ER stress-related proteins were significantly reduced by BAPTA/AM (Fig. 4d). Finally, pretreatment with BAPTA/AM reduced silibinin-induced apoptosis (Fig. 4e) and inhibited the expression of apoptosis-related proteins, such as pro-caspase-3 and PARP (Fig. 4f). These results indicated that Ca^2+^ signaling played an important role in silibinin-mediated ER stress response and apoptosis in PC-3 cells.

### Silibinin induced ROS-mediated Ca^2+^ signaling and ER stress response

**Fig. 5:**
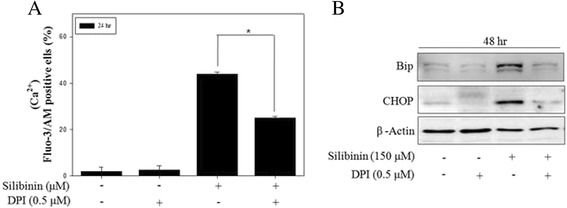
Mitochondrial ROS mediated silibinin-induced ER stress in PC-3 cells. **a** PC-3 cells were treated with 150 μM silibinin for 24 h with the presence or absence of 0.5 μM DPI. The intracellular Ca^2+^ concentration was determined by the fluorescence of fluo-3/AM. **b** The effects of DPI on silibinin-induced ER stress were measured by western blotting. β-Actin was used as a loading control. Data are presented as mean ± SD (*n* = 3 in each group). ^*^ *p* <0.001 vs. the control group.

Previous studies suggested that ROS are an important component leading to UPR in the ER and ER stress-triggered apoptosis [34, 35]. To examine whether ROS are correlated with the silibinin-induced ER stress, intracellular Ca^2+^ flux and ER stress-related proteins were observed after pretreatment with DPI. The pretreatment with DPI reduced the intracellular Ca^2+^ levels and the expression of Bip and CHOP in silibinin-treated PC-3 cells (Fig. 5a and b). These results indicated that downstream targets of mitochondrial ROS are both Ca^2+^ signaling and ER stress response, probably caused by mitochondrial dysfunction. These data strongly suggested that mitochondrial ROS production by silibinin was essential for induction of ER stress via Ca^2+^ signaling pathway and ultimately led to apoptosis in PC-3 cells.

## Discussion

Currently, more effective strategies must be necessary to develop novel therapeutic targets and molecular regulatory agents for cancer diseases. Recently, the role of phytochemicals has been suggested in various cancer managements, especially polyphenolic compounds [36]. Silibinin isolated from milk thistle is a polyphenolic flavonoid and it has been studied for applications as anticancer agents [37]. In addition, some report proposed that silibinin suppresses the tumor growth and exhibits anti-proliferative, pro-apoptotic and anti-angiogenic effects of dietary feeding of silibinin on PC-3 xenograft in vivo [38]. However, complete mechanism on anticancer effect of silibinin has not been clearly identified until now. To further elucidate the silibinin-induced intracellular mechanism, we investigated the various phenomena involved in cell death such as ROS and ER stress response.

First, to investigate silibinin induce cancer selectively death mechanism, apoptosis and ROS were measured in prostate cell lines including androgen-independent PC-3, androgen-dependent LNCaP and normal prostate epithelial RWPE-1 cells. The results indicated that silibinin possesses selective effects on apoptotic cell death and ROS in PC-3 and LNCaP cells, while not affecting the RWPE-1 cells (Additional file 1: Figure S2A and B). ROS are major molecules of intracellular signaling cascades and trigger mitochondria-associated events including apoptosis [39], which is generated from various cellular sources such as NOXs, mitochondrial respiration and extracellular stress. Our results showed that silibinin significantly stimulated the intracellular ROS production in a time-dependent manner. Among various cellular sources of ROS production, NOX4 produces ROS of superoxide type in the mitochondria. In our system, whereas early ROS production by silibinin was inhibited by all ROS inhibitors, persistent and excessive ROS production were attenuated by only DPI treatment, suggesting that silibinin mainly stimulated NOX4-dependent ROS of superoxide type. Also, we observed the mitochondrial ROS production and mRNA expression of the variety of NOX isoforms in silibinin-treated PC-3 cells. NOX plays an important role in regulation of mitochondrial ROS. Also it was reported that NOX4 is localized at various subcellular compartments such as mitochondria, ER, plasma membrane and nucleus [40–42]. Our results showed that silibinin-stimulated ROS were colocalized with NOX4 in mitochondrial portion confirmed by MitoSOX. Expression of NOX4 increased by silibinin was suppressed by DPI, a specific NOX inhibitor. Although some studies have reported that DPI inhibits NOX activity, like our results, another reported that DPI inhibited NOX4 expression in PMA-stimulated U87MG cells [43]. In addition, DPI inhibited silibinin-induced apoptosis and the expression of apoptosis proteins. These results suggested that NOX4 are the primary source of silibinin-stimulated mitochondrial ROS production and these ROS are involved in apoptosis process by modulating NOX4-driven ROS generation from mitochondria.

Some studies reported that intracellular Ca^2+^ flux also mediates multiple cellular signaling cascades of survival and apoptosis. Release of Ca^2+^ from ER and high Ca^2+^ concentration in cytosol trigger cell death in human cancer cell lines [44]. Our results indicated that silibinin markedly increased the level of intracellular Ca^2+^ up to 24 h after treatment. And these effects were prevented by pretreatment with Ca^2+^ chelator BAPTA/AM. Also BAPTA/AM inhibited silibinin-induced apoptosis and the expression of apoptosis proteins. From these results, silibinin provoked the disruption of Ca^2+^ homeostasis, leading to cellular apoptosis. Also Ca^2+^ signaling is considered as one of the second messengers strongly involved in ER stress response [45]. ER stress occurs due to the accumulation of unfolded proteins and disruption of Ca^2+^ homeostasis, and triggers specific signaling pathway with UPR through three key proteins, including IRE1α, ATF4 and PERK. Silibinin induced the expression of ER stress proteins and spliced XBP1 mRNA. In addition, pretreatment with BAPTA/AM markedly decreased the expression of Bip and CHOP. So far, 14 caspase family members have been identified [46] and play important roles in ER stress and apoptosis. Silibinin-induced apoptosis was reduced by Z-YVAD-FMK, an inhibitor of caspase-4, which is localized in the ER membrane (Additional file 1: Figure S3). These results indicated that ER stress plays major role in silibinin-induced apoptosis through Ca^2+^ signaling. On the observation to further clarify the role of ROS in Ca^2+^ flux and ER stress, blocking of ROS production with DPI caused the reduction of Ca^2+^ level and inhibition of ER stress proteins. It was suggested that ROS generation by silibinin plays a key role in induction of ER stress triggered from disruption of Ca^2+^ homeostasis in PC-3 cells.

## Conclusions

In conclusion, our studies demonstrated that silibinin induced apoptosis mediated by activation of ER stress that requires the disruption of Ca^2+^ homeostasis through ROS generation and those ROS were produced, in part, from mitochondrial NOX system, upstream pathway of Ca^2+^ signaling. Therefore, based on these results, regulation of NOX4-driven mitochondrial ROS production could be a potential target for development of the cancer therapy and management.

## Abbreviations

ATF6, activating transcription factor 6; BAPTA, 1,2-bis-(o-Aminophenoxy) ethane-tetraacetic acid tetra-(acetoxymethyl) ester; DAPI, 6-Diamidino-2-phenylindole dihydrochloride; DCFH-DA, 2′,7′-dichlorfluorescein-diacetate; DPI, diphenyleneiodonium (DPI); eIF2α, Eukaryotic initiation factor 2; ER, endoplasmic reticulum; Fluo-3/AM, 4-(6-Acetoxymethoxy-2,7-dichloro-3-oxo-9-xanthenyl)-4′-methyl-2,2′(ethylenedioxy)dianiline-N,N,N′,N′-tetraacetic acid tetrakis (acetoxymethyl) ester; IRE1, inositol requiring enzyme 1; MMP, mitochondrial membrane potential; MTT, 3-(4,5-Dimethyl-thiazol-2-yl)-2,5-diphenyltertrazolium bromide; NOX, NADPH oxidase; PERK, pancreatic ER kinase (PKR)-like ER kinase; PI, propidium iodide; ROS, reactive oxygen species; UPR, Unfolded protein response; XBP1, X box-bonding protein 1

## Additional file

Additional file 1: Figure S1. Silibinin reduced cell viability and stimulated ROS production in human prostate cancer PC-3 cells. (A) PC-3 cells were incubated with silibinin for indicated concentrations and times. Cell viability was determined by MTT assay as described in Materials and Methods. (B) PC-3 cells were treated with 150 μM silibinin for 3 h with the presence or absence of 0.5 μM DPI, 5 mM NAC, 10 μM Tempol and 100 U/ml CAT and ROS production was determined by the fluorescence of DCFH-DA with flow cytometry. Data are presented as mean ± SD (*n* = 3 in each group). #*p* <0.05, ¶*p* <0.01, **p* <0.001 vs. the control group. **Figure S2.** Silibinin induced apoptosis and ROS production selectively in prostate cancer cells but not in normal cells (A) Apoptosis of prostate cell lines was analyzed after treatment with indicated silibinin concentration for 48 h by flow cytometry. (B) Prostate cell lines were treated with indicated silibinin concentration for 48 h. ROS production was determined by the fluorescence of DCFH-DA with flow cytometry. Data are presented as mean ± SD (*n* = 3 in each group). #*p* <0.05, ¶*p* <0.01, **p* <0.001 vs. the control group. **Figure S3.** Silibinin induced ER-dependent apoptosis in PC-3 cells. Inhibition of apoptosis by Z-YVAD-FMK, a caspase-4 inhibitor, was analyzed after treatment with 150 μM silibinin for 48 h with the presence or absence of 5 μM Z-YVAD-FMK by flow cytometry. Data are presented as mean ± SD (*n* = 3 in each group). ^*^ *p* <0.001 vs. the control group. (PPTX 720 mb)
