# Supplementary material for: Silibinin induces mitochondrial NOX4-mediated endoplasmic reticulum stress response and its subsequent apoptosis
Source: BMC Cancer. 2016 Jul 12;16:452. doi: 10.1186/s12885-016-2516-6 (PMC4942927; doi:10.1186/s12885-016-2516-6)
Supplement: Additional file 1: Figure S1. — Silibinin reduced cell viability and stimulated ROS production in human prostate cancer PC-3 cells. (A) PC-3 cells were incubated with silibinin for indicated concentrations and times. Cell viability was determined by MTT assay as described in Materials and Methods. (B) PC-3 cells were treated with 150 μM silibinin for 3 h with the presence or absence of 0.5 μM DPI, 5 mM NAC, 10 μM Tempol and 100 U/ml CAT and ROS production was determined by the fluorescence of DCFH-DA with flow cytometry. Data are presented as mean ± SD (n = 3 in each group). #p <0.05, ¶p <0.01, *p <0.001 vs. the control group. Figure S2. Silibinin induced apoptosis and ROS production selectively in prostate cancer cells but not in normal cells (A) Apoptosis of prostate cell lines was analyzed after treatment with indicated silibinin concentration for 48 h by flow cytometry. (B) Prostate cell lines were treated with indicated silibinin concentration for 48 h. ROS production was determined by the fluorescence of DCFH-DA with flow cytometry. Data are presented as mean ± SD (n = 3 in each group). #p <0.05, ¶p <0.01, *p <0.001 vs. the control group. Figure S3. Silibinin induced ER-dependent apoptosis in PC-3 cells. Inhibition of apoptosis by Z-YVAD-FMK, a caspase-4 inhibitor, was analyzed after treatment with 150 μM silibinin for 48 h with the presence or absence of 5 μM Z-YVAD-FMK by flow cytometry. Data are presented as mean ± SD (n = 3 in each group). * p <0.001 vs. the control group. (PPTX 720 mb) [file 12885_2016_2516_MOESM1_ESM.pptx]

## Slide 1
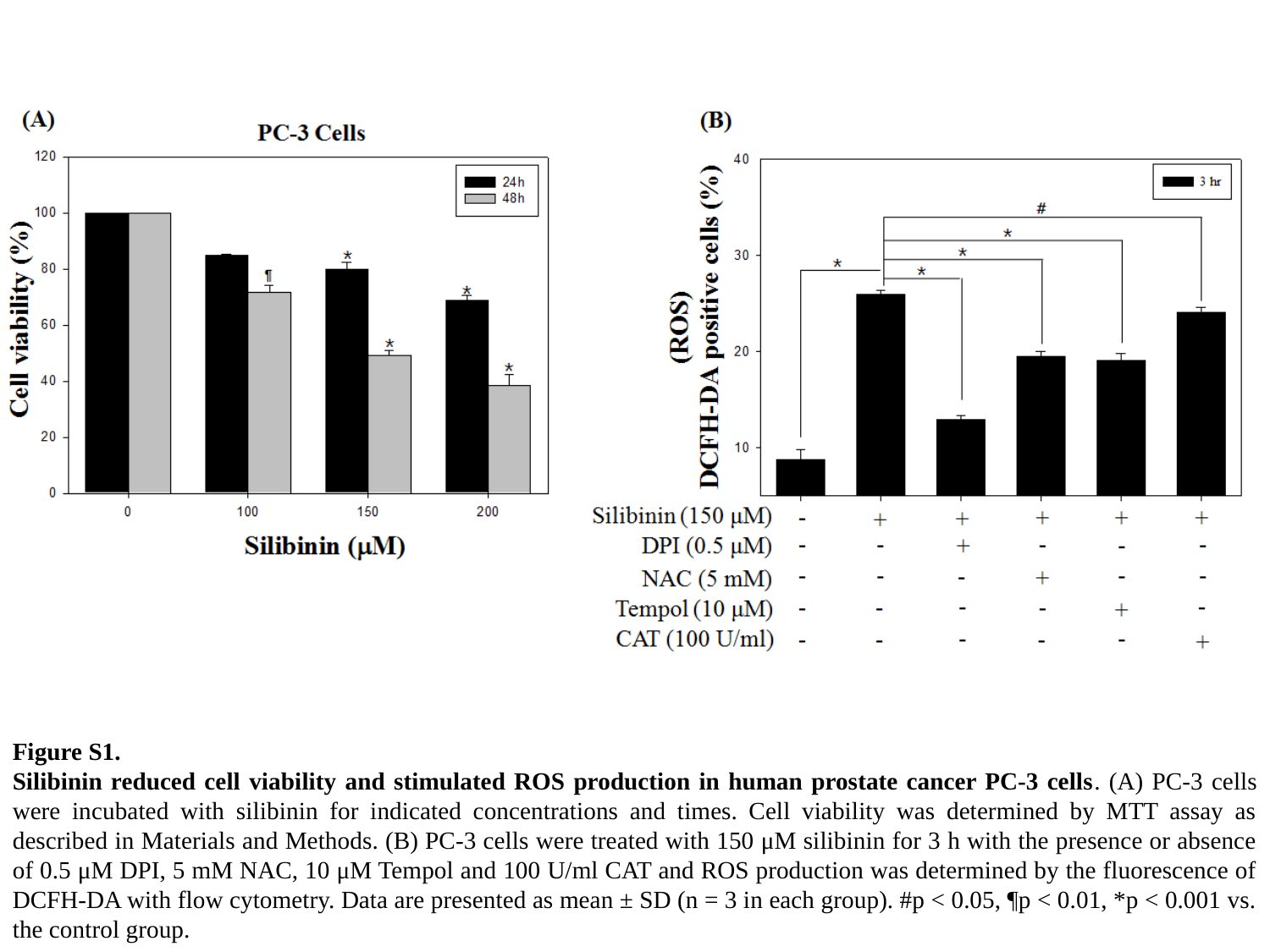

Figure S1.
Silibinin reduced cell viability and stimulated ROS production in human prostate cancer PC-3 cells. (A) PC-3 cells were incubated with silibinin for indicated concentrations and times. Cell viability was determined by MTT assay as described in Materials and Methods. (B) PC-3 cells were treated with 150 μM silibinin for 3 h with the presence or absence of 0.5 μM DPI, 5 mM NAC, 10 μM Tempol and 100 U/ml CAT and ROS production was determined by the fluorescence of DCFH-DA with flow cytometry. Data are presented as mean ± SD (n = 3 in each group). #p < 0.05, ¶p < 0.01, *p < 0.001 vs. the control group.

## Slide 2
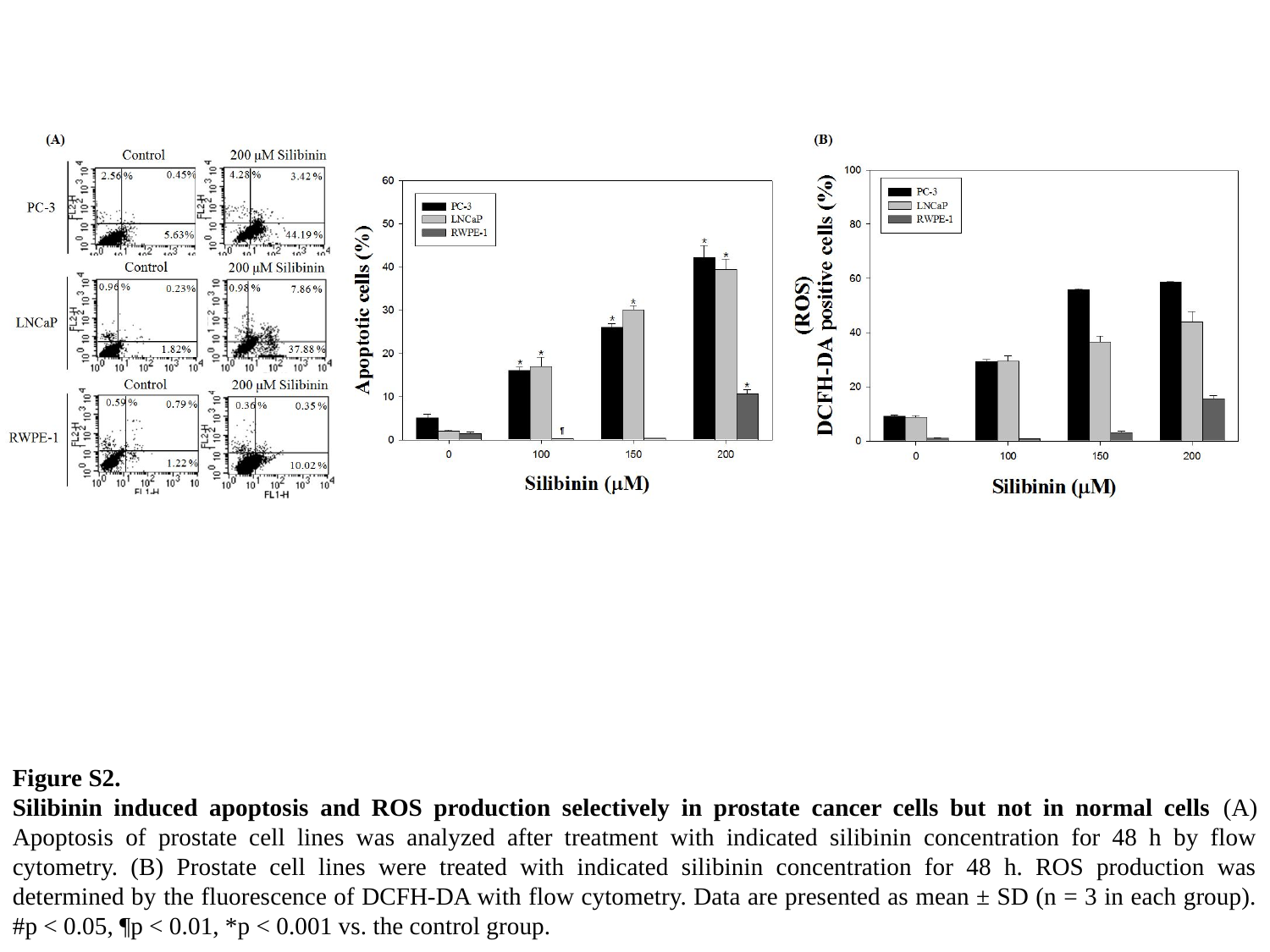

Figure S2.
Silibinin induced apoptosis and ROS production selectively in prostate cancer cells but not in normal cells (A) Apoptosis of prostate cell lines was analyzed after treatment with indicated silibinin concentration for 48 h by flow cytometry. (B) Prostate cell lines were treated with indicated silibinin concentration for 48 h. ROS production was determined by the fluorescence of DCFH-DA with flow cytometry. Data are presented as mean ± SD (n = 3 in each group). #p < 0.05, ¶p < 0.01, *p < 0.001 vs. the control group.

## Slide 3
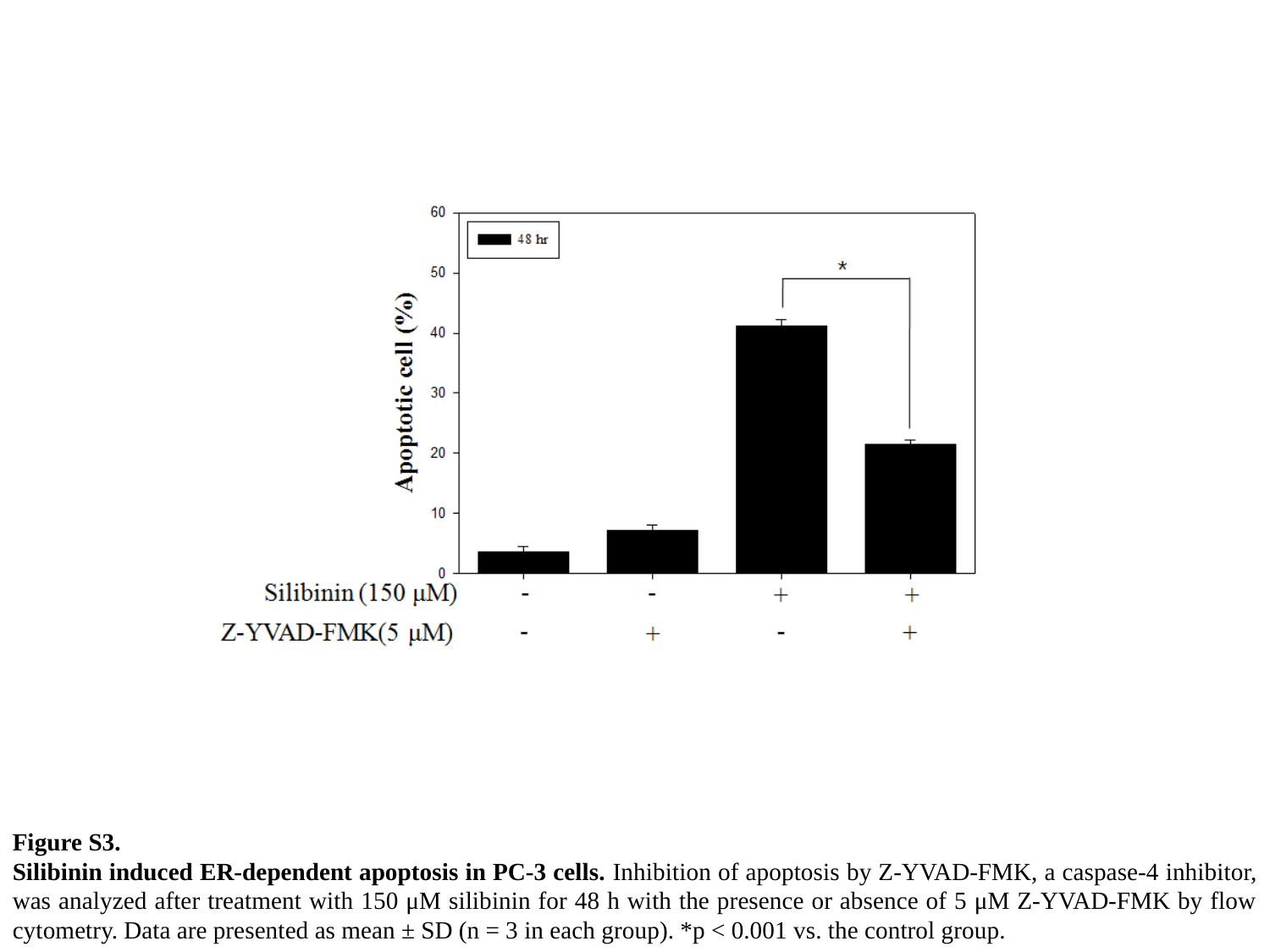

Figure S3.
Silibinin induced ER-dependent apoptosis in PC-3 cells. Inhibition of apoptosis by Z-YVAD-FMK, a caspase-4 inhibitor, was analyzed after treatment with 150 μM silibinin for 48 h with the presence or absence of 5 μM Z-YVAD-FMK by flow cytometry. Data are presented as mean ± SD (n = 3 in each group). *p < 0.001 vs. the control group.
